# Anticancer Mechanisms of Ginsenoside Compound K: A Review

**DOI:** 10.3390/diseases13050143

**Published:** 2025-05-05

**Authors:** Yu-Po Lee, Hui-Ting Chan, Tzu-Hsuan Li, Lichieh (Julie) Chu, Sheau-Long Lee, Yu-Quan Chang, Robert YL Wang

**Affiliations:** 1 Biotechnology Industry Master and Ph.D. Program, Chang Gung University, Taoyuan 333323, Taiwan; jimmylee0719@gmail.com (Y.-P.L.); tinachan@wellhead-bms.com (H.-T.C.); 2 Wellhead Biological Technology Corp., Taoyuan 325019, Taiwan; tiffany91124@gmail.com (T.-H.L.); jameslee@wellhead-bms.com (S.-L.L.); aronchang@wellhead-bms.com (Y.-Q.C.); 3 Graduate Institute of Biomedical Sciences, College of Medicine, Chang Gung University, Taoyuan 333323, Taiwan; julie.chu@mail.cgu.edu.tw; 4 Department of Biomedical Sciences, College of Medicine, Chang Gung University, Taoyuan 333323, Taiwan; 5 Department of Otolaryngology-Head and Neck Surgery, Chang Gung Memorial Hospital, Linkou 333423, Taiwan

**Keywords:** cancer, ginsenoside compound K, stromal cell-derived factor 1, angiogenesis, chronic inflammation

## Abstract

Cancer, also known as malignant tumors, is formed due to abnormal mutations and the proliferation of human cells. Cancer cells not only demonstrate accelerated proliferation but also show robust invasive and metastatic potential, disseminating from a primary affected region of the body to multiple areas and potentially culminating in organ dysfunction or failure, thereby jeopardizing the individual’s life. The rapid growth of the biopharmaceutical market has given rise to numerous novel medicines, thereby precipitating a paradigm shift in contemporary drug development methodologies. This modification is focused on identifying methodologies that can effectively target cancerous cells while minimizing damage to normal cells. There is an increasing societal movement that supports the utilization of natural ingredients derived from plants. In recent years, traditional herbal medicine has experienced a surge in popularity within the global cancer market. In comparison with the use of more toxic chemotherapy methods, there has been an increasing focus on advanced therapies that exhibit reduced side effects. Ginsenoside compound K (CK) is derived from the natural components in ginseng through biotransformation. The utilization of CK in cancer research is a practice engaged in by numerous scientists. The underlying rationale is that CK exhibits a multitude of effects within the realm of cancer research, including but not limited to the mitigation of inflammation, the suppression of cancerous cell proliferation, and the safeguarding of cardiovascular, hepatic, and renal functions. This review methodically identifies and organizes CK-related journals according to the following key points of cancer treatment: the effects on cancer cells themselves, angiogenesis inhibition, modulation of immune response to identify cancer cells, and inflammation regulation. The intricate interplay between ginsenoside CK and cells is elucidated through a graphical representation. The present review focuses on the results of CK in in vitro tests. It is our hope that the present article will aid future studies on the results of CK in vivo tests, clarify the correlation between cellular mechanisms in vivo and in vitro tests, and assist in the development of drugs.

## 1. Introduction

### 1.1. Caner Incidence and Etiology

Cancer is among the foremost causes of mortality on a global scale. According to the International Agency for Research on Cancer (IARC) in its Global Cancer Statistics Report, GLOBOCAN, approximately 19.3 million new cancer cases and around 10 million cancer-related deaths were documented on a global scale in 2020. The IARC anticipates a 47% surge in new cancer cases worldwide from 2020 to 2040. According to the most recent data, it is projected that by the year 2040, the global total of new cancer cases will reach approximately 28.4 million. The most prevalent types of cancer include lung cancer, breast cancer, prostate cancer, and colorectal cancer [1].

The accelerated progression of the biopharmaceutical market has precipitated the formulation of numerous innovative pharmaceuticals, thereby prompting a progressive shift in contemporary drug development methodologies. This evolution is characterized by the pursuit of efficacious cancer cell targeting, while concomitantly ensuring minimal harm to non-cancerous cells. This paradigm shift encompasses the integration of Western medicinal interventions with traditional Chinese medicinal practices, as well as the exclusive utilization of novel, plant-based drug formulations derived from traditional Chinese medicine. There has been a discernible escalation in the awareness of advanced cancer treatments that are characterized by minimal adverse effects. Consequently, traditional herbal medicine has emerged as a prominent trend within the global cancer market in recent years.

The etiology of cancer is multifaceted and varied, and its pathogenesis remains to be elucidated. However, based on previous studies examining the transformation of normal cells into cancer cells, it is understood that exposure to one or more carcinogenic substances can induce fundamental changes in normal cells, including irreparable cytogenetic disorders. Following the transformation of these cells into cancerous ones, their proliferation becomes ungovernable by the body’s regulatory mechanisms. The uncontrolled proliferation of these cells leads to the formation of a malignant tumor [2,3]. In summary, cancer cells exhibit several distinguishing characteristics that set them apart from normal cells. These characteristics include, but are not limited to, the following: (1) genetic mutations; (2) increased proliferative capacity; (3) extrachromosomal DNA (ecDNA) amplification; (4) alterations in telomere length and telomerase activity; (5) enhanced angiogenesis; and (6) increased invasive capacity. A comprehensive overview of these properties and their relationship to cancer can be found in numerous review articles [4,5,6,7,8,9,10,11].

### 1.2. Structural Difference, Chemical and Drug Properties of Ginsenosides

Ginseng has been utilized by numerous scientists engaged in cancer research, in addition to its application in the field of taxonomy, where its genus name, Panax, is derived from the Latin word “Panacea”, signifying “cure-all” [12].

In a review of the previous literature, researcher Wang highlighted that ginsenosides, polysaccharides, and flavonoids are the active constituents of ginseng. Subsequent cell and animal experiments have demonstrated that the active ingredient, “ginsenosides” is a significant source of numerous medicinal properties of ginseng [13].

It is evident that ginseng species, including *Panax notoginseng*, *Panax ginseng*, and *Panax quinquefolius*, are recognized as containing ginsenosides. To date, researchers have identified more than 150 different ginsenosides, which exhibit a wide range of pharmacological properties, including anti-fatigue, anti-inflammatory, immune-modulating, and anti-tumor effects, as well as involvement in the prevention or treatment of cardiovascular and liver/kidney-related diseases [14,15,16,17,18].

**Figure 1:**
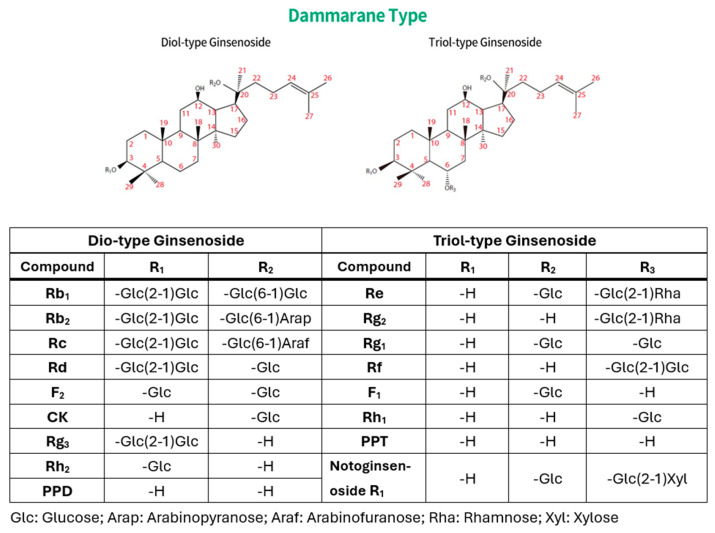
Stereochemical structures of dammarane-type diol and triol ginsenosides. Dammarane has the molecular formula C_30_H_54_ and is a type of tetracyclic triterpene.

**Figure 2:**
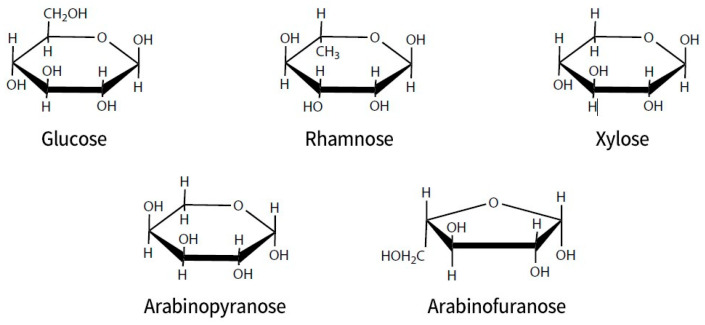
Different glycosyl combinations in ginsenoside structures (including xylose, rhamnose, glucose, arabinofuranose, and arabinopyranose).

There are numerous types of ginsenosides, with current academic research focusing on the dammarane-type diol ginsenosides and triol ginsenosides (Figure 1). The core structure of ginsenosides is aglycone and is almost insoluble in water. It has the capacity to form a variety of diols and triols with different types and quantities of sugar groups. Ginsenosides derived from Panax plants typically contain multiple sugar molecules, and the greater the number of sugar molecules, the greater the solubility in water. Ginsenoside Rb1 contains four sugar molecules attached to its aglycone, resulting in significantly enhanced water solubility relative to ginsenoside Rh2, which contains only one sugar molecule. The removal of sugar molecules results in an almost insoluble compound, referred to as either “propanediol” (PPD) or “propanetriol” (PPT). These compounds are the diol and triol ginsenosides previously referenced in this paragraph. The sugar molecules typically form glycosidic bonds with the hydroxyl groups at the C3 or C20 position of protopanaxadiol (PPD) and at the C6 and C20 positions of protopanaxatriol (PPT). The classification of PPT-type ginsenosides as “triol-type” is attributed to the presence of an additional hydroxyl group at the C3 position. Triol-type ginsenosides have three hydroxyl groups. The hydroxyl group has the capacity to undergo replacement by a hydrogen atom from a sugar, thereby forming a glycosidic bond with oxygen. The glycosyl group of ginsenoside can include xylose, rhamnose, glucose, arabinose, and arabinopyranose (see Figure 2).

The pharmacological properties of ginsenosides are significantly affected by the variation in binding sites (C3, C6, and C20) and types of glycosyl groups. Fischer projection rules can be used to distinguish the stereostructures of ginsenosides with the same molecular weight when they are stereoisomers. Almost every ginsenoside has two stereoisomeric forms at the C20 position: the clockwise R form and the counterclockwise S form. The predominance of the S configuration in naturally occurring ginsenosides at the C20 position has led to the adoption of this configuration as the standard for research material in most studies. The research outcomes demonstrate that the distinct stereostructures of ginsenosides at C20 result in varied pharmacological effects. This is attributable to the fact that the degree of fit between enzymes and substrates, in accordance with the lock and key principle, is a pivotal factor in determining the binding of enzymes to substrates. The activation of different types and levels of enzymes by ginsenosides in the body leads to different physiological responses, as evidenced by the observation that the S configuration of ginsenoside Rh2 demonstrates superior inhibition of prostate cancer cells compared to the R configuration [19]. However, in the context of bone formation, the R configuration of ginsenoside Rh2 selectively inhibits osteoclast proliferation without toxicity, whereas the S configuration shows a weak inhibition of osteoclast proliferation and significant toxicity [20].

### 1.3. The Biological Metabolism of Ginsenosides, the Chemical and Drug Properties of Ginsenoside Compound K

**Figure 3:**
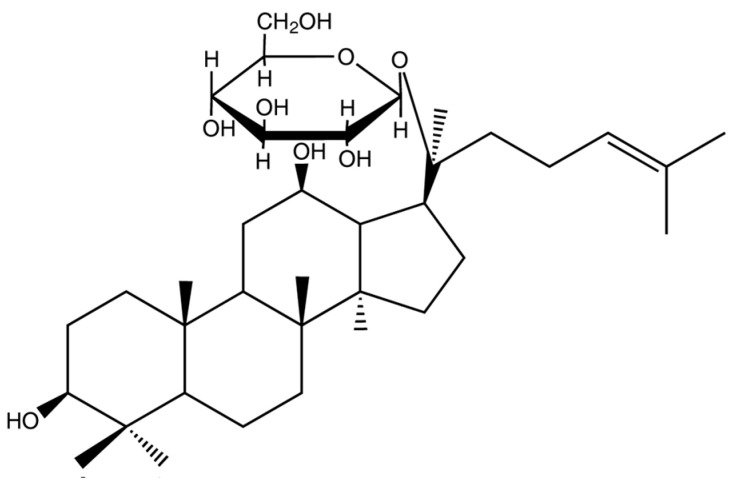
The chemical structure of ginsenoside compound K (CK).

Recent advances in scientific research have facilitated the analysis and identification of the manner in which a component manifests at varying stages of the digestive process within the human body. The majority of ingested substances undergo a transformation into smaller molecules for the purpose of absorption following gastrointestinal processing. Natural ginsenosides, which are found in plants belonging to the Panax genus, typically carry 3 to 4 sugar groups. However, following degradation by gastrointestinal fluids and metabolic transformation by gut microbiota, these ginsenosides are converted into forms with only 1 to 2 sugar groups before being absorbed into the bloodstream through the small intestine [21]. Ginsenoside Rg3, Rh2, and compound K (CK) (see Figure 3) are products that, following digestion in the gastrointestinal tract, contain only 1 to 2 sugar groups.

A number of experiments have demonstrated the efficacy of these three rare ginsenosides in the inhibition of inflammation and cancer [22,23]. However, under typical conditions of oral ginseng administration, it is challenging to achieve a sufficient absorption of these rare ginsenosides after intestinal transformation for therapeutic use within the body. Research has demonstrated that in red ginseng sourced from New Zealand, the contents of ginsenoside Rg3 and Rh2 per gram of the dried ginseng sample are only 0.001% and 0.003%, respectively [24]. Furthermore, ginsenoside CK is not a naturally occurring compound in ginseng plants, as it is exclusively derived from gastrointestinal degradation and microbial metabolism. In 1972, Dr. Kitagawa, a Japanese researcher, published a study demonstrating that soil bacteria can degrade natural ginsenosides from ginseng to produce CK in vitro. According to the IUPAC organic compound naming conventions, ginsenoside CK is defined as 20-O-β-D-glucopyranosyl-20(S)-protopanaxadiol [25]. However, despite the potential for ginsenoside CK formation through gastrointestinal degradation and microbial transformation in vivo, research by Hasegawa in 1996 indicated that rats fed a total ginsenosides dose of 1 g/kg/day showed only a 0.001% conversion rate of CK in their blood after 24 h [21].

It is thus the case that the mere ingestion of ginseng may not result in the achievement of effective therapeutic levels regarding the treatment of diseases. Consequently, a future trend involves the artificial simulation of the in vivo conversion process to produce stable quantities of ginsenoside CK. It is noteworthy that ginseng plants themselves do not inherently contain ginsenoside CK. The prevailing methodologies for artificial production encompass chemical synthesis, microbial transformation, and enzymatic transformation [26]. Among these methods, microbial transformation remains a predominant mode of stable production. The method under consideration entails the utilization of solid-state fermentation as a means to convert total ginsenoside extracts, comprising ginsenoside Rb1, Rb2, Rb3, Rc, and other constituents, into the rare ginsenoside CK.

Ginsenoside CK has demonstrated significant potential in the prevention and treatment of various diseases, owing to its multifaceted pharmacological actions, including anti-inflammatory, anticancer, immune-modulating, anti-angiogenic, neuroprotective, hepatoprotective, nephroprotective, and cardiovascular-protective properties [16,18,22]. For instance, ginsenoside CK has been shown to induce apoptosis in tumor cells, thereby inhibiting tumor growth [27]. This article also provides a synopsis of the IC_50_ values of CK for various cancer cells. Our findings indicate that the IC_50_ values of glioma and neuroblastoma range from 3 to 15 μM, exhibiting heightened sensitivity to CK compared to other cancer cell types. As illustrated in Table 3, the substance has been shown to modulate the tumor microenvironment by targeting proteins implicated in angiogenesis, thereby reducing immunosuppressive cells, such as myeloid-derived suppressor cells (MDSCs) to enhance immune response and inhibit tumor metastasis [27]. In a 2019 study, Hsu et al. demonstrated that ginsenoside CK modulates mitochondrial-related cellular signaling pathways in renal cells and inhibits the activity of the NLRP3 inflammasome, thereby reducing tubulointerstitial damage and offering protection and treatment for kidneys [18]. The capacity of ginsenoside CK to modulate NLRP3 is central to its anti-inflammatory properties, which are of particular significance given that inflammation is frequently the underlying cause of numerous diseases.

Most anticancer drugs cause side effects like rash, nausea, vomiting, hair loss, oral mucosal inflammation, abnormal skin sensation, taste changes, and bone marrow suppression. A summary of the “no observed adverse effect level (NOAEL)” doses from several toxicity studies is provided in Table 1 [28,29,30]. A comparison of animal models of different cancer types is also presented in Table 2 [31,32,33,34,35,36,37]. Our findings indicate that the effective therapeutic doses employed in various mouse models of cancers are within the NOAEL. At present, the NOAEL of ginsenoside CK and the effective therapeutic dose for cancer in humans have not been published in any journal. Therefore, it can be hypothesized that ginsenoside CK exhibits minimal toxicity within the effective dose range when administered to these animal models.

Ginsenoside CK, in particular, has a molecular weight of 622 Da, demonstrating strong hydrophobicity properties, minimal toxicity at effective therapeutic doses in various cancers in animal models, remarkable anti-inflammatory properties, and the capacity to effectively traverse the blood–brain barrier [38]. These characteristics position ginsenoside CK as a promising lead compound for drug development. The general routes of administration encompass various methods, including eye drops, skin application, sublingual absorption, oral administration, subcutaneous injection, peritoneal injection, intramuscular injection, and intravenous injection, among others. Hydrophilic substances are known to exhibit high biocompatibility, with the capacity to mitigate tissue irritation and inflammatory responses when in contact with the human body. Ginsenoside CK is characterized by its extreme hydrophobicity, which precludes its dissolution in water-soluble preparations. Consequently, researchers frequently employ excipients to enhance the solubility of active principles. Excipient types may include surfactants, glidants, disintegrants, binders, buffers, colorants, flavorings, antioxidants, preservatives, and film-forming agents. Among them, excipients with surfactant properties, such as polyethylene glycol (PEG), have been shown to enhance the solubility of CK in water-soluble preparations. Presently, the predominant administration modalities of CK in non-clinical trials are oral and intravenous injection [28,29,30], while only oral trials are available in clinical trials [39]. The synthesis of ginsenoside CK is challenging, and the cost is significant. In the event that CK is administered orally, a significant portion of the substance may be lost following digestion and metabolism within the gastrointestinal tract. Therefore, in order to maintain a therapeutic dose, it may be more appropriate to administer CK preparations intravenously.

In recent years, a significant number of academic journals have explored the application of various ginsenosides in cancer treatment. This review article focuses on investigating the anticancer mechanisms of ginsenoside CK, a protopanaxadiol-type ginsenoside. Recognizing ginsenoside CK’s effective inhibition of cancer cells through diverse cellular mechanisms, this review paper consolidates findings from international literature to propose ten mechanisms by which ginsenoside CK inhibits cancer. Despite the proliferation of studies on CK, a comprehensive review that consolidates its molecular mechanisms against cancer remains lacking. Furthermore, the objective of this review is to elucidate the pharmacological mechanisms of action of ginsenoside CK by graphically illustrating these complex interactions, with the aim of demonstrating its dramatic effect on suppressing cancer cells. It is hypothesized that in consideration of the effects of CK on cancer cell proliferation, migration, invasion and angiogenesis of tumor tissues, as well as immune regulation, researchers in the field of drug development will be able to further understand the overall differences caused by CK in cancer in non-clinical and clinical outcomes through these related microscopic mechanisms.

## 2. Different Mechanisms of Ginsenoside CK Targeting Cancer Therapy

### 2.1. Ginsenoside CK Inhibits Cancer Cell Proliferation and Invasion by Reducing the Activity of PI3K/AKT/mTOR/p70S6K1 Signaling Pathway Proteins

**Figure 4:**
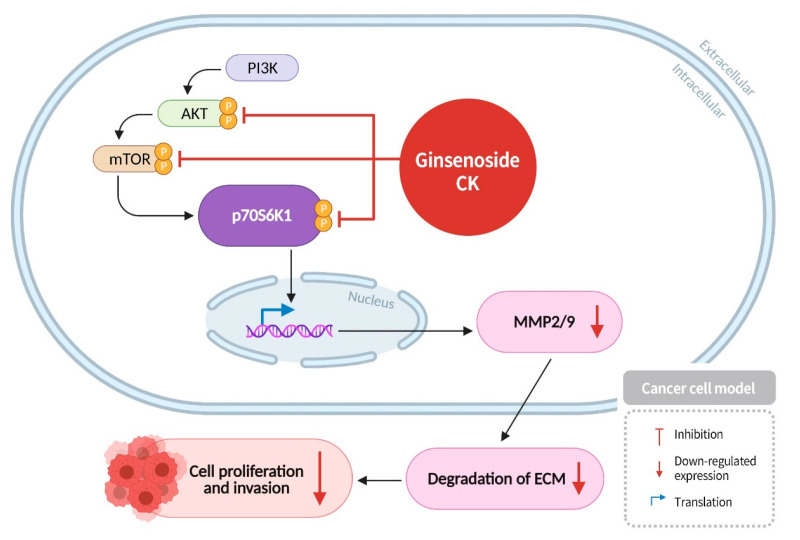
Ginsenoside CK inhibits the expression of AKT/mTOR/p70S6K1 proteins and blocks the PI3K/AKT/mTOR signaling pathway in cancer cells, reducing the expression of MMP2 and MMP9 proteases. This results in the inhibition of cancer cell proliferation and invasion.

The PI3K/AKT/mTOR pathway is a protein kinase cascade within cells that collectively regulates processes such as cell growth, proliferation, apoptosis, angiogenesis, and autophagy, playing a pivotal role in important signaling pathways [40]. Aberrant activation of this pathway in normal cells can lead to cellular transformation, neurodegenerative diseases, or autoimmune disorders [41]. In cancer cells, activation of this protein kinase cascade promotes proliferation, invasion, and metastasis. PI3K plays a crucial role in cell metabolism, proliferation, and movement, while AKT, regulated mainly by PI3K, modulates cell survival, proliferation, migration, and glucose metabolism. mTOR also acts as a regulatory factor, primarily promoting protein synthesis, cell proliferation, and energy metabolism. In the context of cancerous cells, mTOR dysfunction has been shown to result in uncontrolled proliferation and impaired differentiation [42]. p70S6K1, a pivotal regulator of cell growth, survival, and metabolism, has also been identified as a factor in the invasion and metastasis of cancer cells [43]. Matrix metalloproteinases (MMPs), a class of proteases that degrade the extracellular matrix, have been demonstrated to play a critical role in cancer cell migration and invasion [43]. Research has indicated that PI3K in cells can induce AKT activation through phosphorylation pathways, and phosphorylated AKT subsequently activates mTOR, which in turn phosphorylates and activates p70S6K1 [44]. Activated p70S6K1 has been shown to promote the expression of MMP2/9, leading to the degradation of the extracellular matrix and facilitating cancer cell migration. The activation of the PI3K/AKT/mTOR signaling pathway in cancer cells has been shown to promote cancer cell proliferation and invasion [45]. Research has found that ginsenoside CK can effectively inhibit the expression of AKT/mTOR/p70S6K1 proteins in cancer cells, block the PI3K/AKT/mTOR signaling pathway, and reduce the expression of MMP2 and MMP9 proteases. This inhibition results in reduced cancer cell proliferation and invasion, ultimately inducing cancer cell apoptosis (Figure 4) [46].

### 2.2. Ginsenoside CK Can Reduce the Activity of Stromal Cell-Derived Factor 1 (SDF-1), Thereby Inhibiting the Migration of Cancer Cells

**Figure 5:**
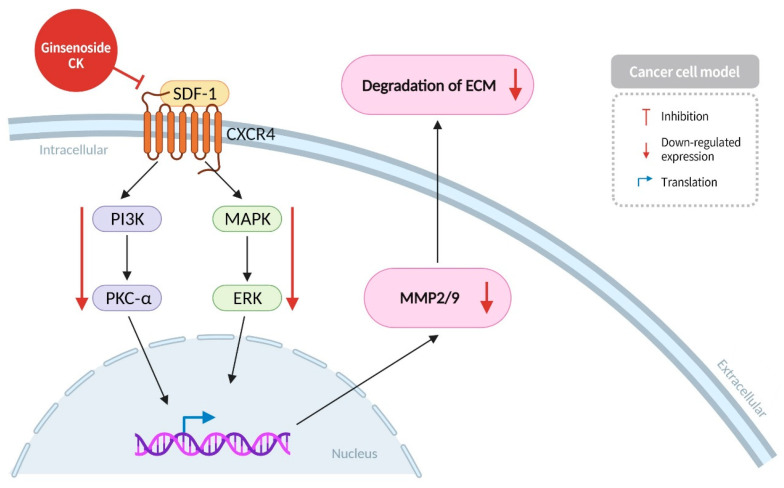
Ginsenoside CK reduces the binding of SDF-1 to CXCR4, thereby inhibiting the activation of PKC-α and ERK, resulting in a decreased expression of MMP2 and MMP9 proteases downstream in the signaling pathway.

Cancer cell metastasis is a critical process in cancer development, and the ability of cancer cells to migrate from their primary site (primary tumor) to other parts of the body (secondary tumors) is a key factor in the complexity of treatment and the potential for severe outcomes. This process is the initial stage of cancer cell metastasis, and the ability to block cancer cell migration at this stage can effectively inhibit cancer progression. SDF-1, a critical factor in cancer cell migration, plays a pivotal role in various pathological and physiological mechanisms, including embryonic development, wound healing, and angiogenesis [47]. In cancer cells, the binding of SDF-1 to CXCR4 activates processes such as angiogenesis, invasion, and migration. The PI3K/PKC-α axis plays a pivotal role in regulating diverse processes such as cancer cell growth, apoptosis, and migration, thereby contributing to the progression of cancer [48]. MAPKs (mitogen-activated protein kinases) are ubiquitously present in cells, and ERK (extracellular signal-regulated kinases) is capable of transmitting extracellular signals to the cytoplasm and nucleus. Its primary functions encompass the regulation of gene expression, cell differentiation, and proliferation. The MAPK/ERK pathway is the most significant among all MAPK signaling pathways, playing a crucial role in the survival and development of tumor cells [49]. After the binding of SDF-1 with CXCR4, the signal for cell migration is transmitted to PI3K and MAPKs. This leads to the phosphorylation and activation of PKC-α and ERK, respectively. This activation increases the expression of MMP2/9 proteins, promoting ECM degradation and thereby facilitating cancer cell migration. Research findings demonstrate that ginsenoside CK reduces the binding of SDF-1 to CXCR4, thereby inhibiting the activation of PKC-α and ERK. This reduction leads to a decreased expression of downstream MMP2 and MMP9 proteases, which helps prevent cancer cell migration and metastasis (Figure 5) [50].

### 2.3. Ginsenoside CK Inhibits Angiogenesis in Cancer Cells and Blocks Cancer Cell Growth

The concept of cancer angiogenesis, as postulated by Folkman in 1971 in *the New England Journal of Medicine*, posits that the survival of cancer cells is contingent on the process of angiogenesis, which facilitates the delivery of oxygen and nutrients. In the nascent stages of tumor formation, cancer cells can subsist by extracting nutrients from their immediate environment through diffusion. However, as the cancer cells expand to a size larger than 0.3 cm, they necessitate a greater supply of oxygen and other essential nutrients. To achieve this, the cancer cells themselves or the surrounding connective tissue secrete substances that promote angiogenesis, such as basic fibroblast growth factor (bFGF) and vascular endothelial growth factor (VEGF). By inducing angiogenesis, the cancer cells meet the increased demand for growth. Therefore, if angiogenesis in cancer cells can be blocked, the nutrient supply to the cancer cells can be reduced, leading to the cessation of their growth, shrinkage, or even cell death.

**Figure 6:**
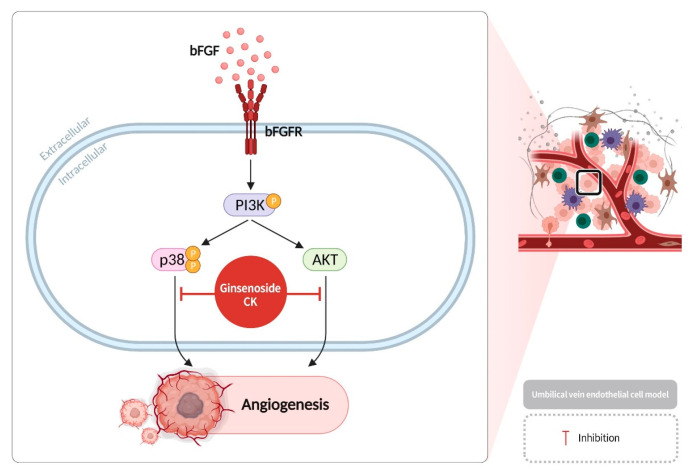
Ginsenoside CK can inhibit the p38 and AKT signaling pathways downstream of PI3K in endothelial cells, blocking angiogenesis near cancer tissues.

Angiogenesis is a series of processes involving complex interactions between various biological components, including different types of cells, soluble angiogenic factors, and the extracellular matrix [51]. The process comprises four distinct sequential steps: a. Proteolytic enzymes degrade the basement membrane glycoproteins and other extracellular matrix components surrounding blood vessels. b. Endothelial cells are activated and migrate. c. Endothelial cells proliferate. d. Endothelial cells transform into tubular structures, forming capillaries, and simultaneously develop a new basement membrane [45,52]. This process enables new capillaries to emerge from the pre-existing vascular network, thereby supplying the requisite oxygen and nutrients to the tissue [53]. Angiogenesis, by contrast, typically occurs during embryonic development, the female reproductive cycle, and wound healing [54]. However, cancer cells have been observed to induce abnormal angiogenesis by secreting growth factors. bFGF (basic fibroblast growth factor) is a notable example of a growth factor that can promote cell division and survival when it binds to its receptor, bFGFR (basic fibroblast growth factor receptor). It is involved in embryonic development, cell growth, tissue repair, cancer cell proliferation, and invasion in the human body [55]. PI3K plays a significant role in cell metabolism, proliferation, and migration [56,57].The p38 signaling pathway promotes cell survival and inhibits apoptosis, playing a crucial role in cell proliferation and apoptosis, epithelial–mesenchymal transition, and tumor invasion [58]. AKT regulates cell survival, proliferation, migration, and glucose metabolism. Upon binding to its receptor bFGFR on cancer cells, bFGF activates PI3K, inducing p38 phosphorylation and thereby activating the AKT signaling pathway, thus promoting angiogenesis around the cancer cells. Studies have demonstrated that bFGF stimulates angiogenesis in human umbilical vein endothelial cells (HUVECs) [59]. Ginsenoside CK has been shown to inhibit the p38 and AKT pathways downstream of PI3K, thereby blocking the formation of new blood vessels and exerting an inhibitory effect on cancer cells (Figure 6) [60].

### 2.4. Ginsenoside CK Inhibits Human Telomerase Reverse Transcriptase (hTERT) and Telomerase Activity, Inducing Apoptosis in Cancer Cells

**Figure 7:**
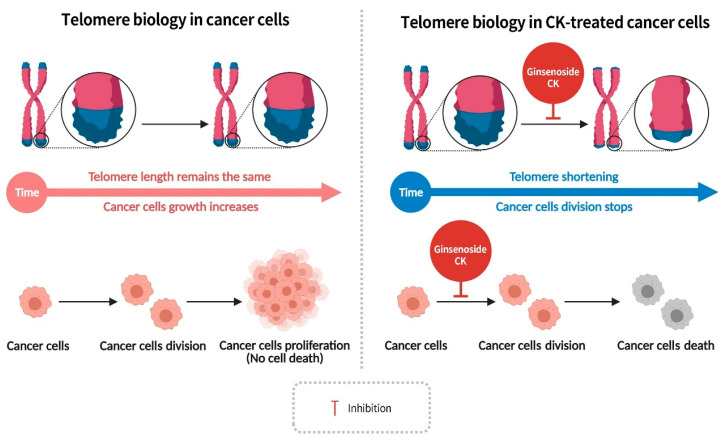
Ginsenoside CK inhibits the activity of telomerase reverse transcriptase and telomerase in cancer cells, shortening telomere length and halting cell division, inducing apoptosis.

Telomeres are DNA repeat sequences located at the extremities of eukaryotic chromosomes, and it has been established that during normal cell division, these structures shorten by 25 to 200 base pairs each time. As these repeat units progressively decrease with each division, the length of the telomeres is reduced. When these structures shorten to a critical length that is unable to maintain chromosome stability, cells initiate a process known as apoptosis, a crucial mechanism for maintaining metabolic balance in organisms. In contrast, cancer cells maintain their telomere length by increasing telomerase activity, thereby enabling uninterrupted division without undergoing cell death. Consequently, reducing telomerase activity in cancer cells can arrest cell division, promote cellular senescence, and induce apoptosis. Telomeres are composed of non-coding repetitive sequences of bases (5′-TTAGGG-3′). In humans, the length of telomere segments ranges between 5000 and 15,000 base pairs. This extensive stretch of repetitive DNA sequences is characterized by a single-stranded overhang structure at the 3′ end, which is inserted into the chromosome ends, forming a structure known as the T-loop [61]. During cell division, telomeres undergo a process of shortening, and the task of restoring these repetitive sequences to the ends of chromosomes is shouldered by telomerase, a ribonucleoprotein complex and a type of reverse transcriptase. The function of telomerase is twofold: firstly, it prevents cells from undergoing apoptosis due to telomere shortening, and secondly, it plays a crucial role in regulating the cell cycle and maintaining chromosome integrity [62]. Human telomerase is composed of a reverse transcriptase and RNA. Human telomerase reverse transcriptase (hTERT) utilizes telomerase RNA as a template to synthesis DNA, thereby elongating the telomeres at the ends of chromosomes [63]. In cancer cells, the concentration of hTERT is significantly higher compared to normal cells, enabling cancer cells to maintain telomere length [64]. Consequently, inhibitors of human telomerase reverse transcriptase represent important targets for cancer therapy. Research has demonstrated that compound K impedes the activity of hTERT and telomerase in cancer cells, resulting in telomere shortening and the cessation of cancer cell division, thereby inducing apoptosis (Figure 7) [62]. This suggests that compound K possesses potential as a cancer therapeutic agent.

### 2.5. Ginsenoside CK Can Reduce Chronic Inflammation Caused by Excessive Inflammatory Responses and Prevent Cell Carcinogenesis

The inflammatory response is a multifaceted phenomenon in human health, exhibiting both beneficial and detrimental aspects. Under normal circumstances, inflammation is a physiological response that occurs during tissue repair or as a response to bacterial or viral infection. Prolonged inflammation that persists without any external threats is classified as chronic inflammation. Inflammation is primarily categorized into two types: acute and chronic. Recent studies have indicated that acute inflammation may exhibit anticancer effects in cancer development, whereas chronic inflammation can lead to various pathologies, including atherosclerosis, rheumatoid arthritis, fibrosis, and even cancer. It is widely accepted that chronic inflammation, when present over an extended period, can exhaust immune cells, thereby reducing the body’s immunity and allowing cancer cells to evade immune surveillance. This, in turn, can increase their growth potential and lead to cancer. Inflammatory responses have been demonstrated to influence cancer progression by regulating cellular behaviors, multiple signaling pathways, and immune responses [49]. Many studies use LPS to induce inflammatory responses in macrophages. LPS binds to TLR4 on macrophages, thereby activating the IKK complex and MAPKs. The MAPKs include three family members: ERK, JNK, and p38, which can induce NF-κB activation to regulate gene expression, cell differentiation, and proliferation processes, among many other biochemical processes [49]. COX-2 and iNOS are two important proteases produced by NF-κB target genes, which, respectively, produce PGE2 and NO. NO is a critical signaling molecule, and its expression can lead to inflammation and tissue damage; PGE2 increases cancer cell proliferation and invasion, promoting metastasis and angiogenesis. Therefore, the occurrence of inflammatory responses plays a critical role in cancer progression [65].

**Figure 8:**
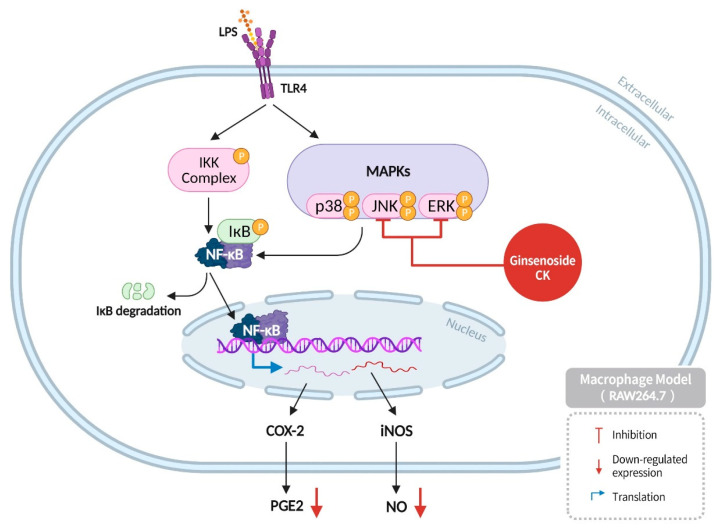
Ginsenoside CK can inhibit the inflammatory response induced by lipopolysaccharides, reduce the expression levels of ERK and JNK, and decrease the production of inflammation causing NO and PGE2.

Studies have found that ginsenoside CK can inhibit the inflammatory responses induced by lipopolysaccharide, reduce the expression of ERK and JNK, and decrease the production of NO and PGE2, which cause cellular inflammation. Consequently, this prevents cells from becoming cancerous due to prolonged exposure to an inflammatory environment (Figure 8) [66].

### 2.6. Bidirectional Regulation of Macrophage Immune Activity by Ginsenoside CK to Inhibit Cancer Cell Proliferation

Macrophages, a type of innate immune system cell, are distinguished by their ability to rapidly recognize pathogens and foreign substances, thus playing a pivotal role in the maintenance of bodily homeostasis. The innate immune system constitutes the body’s intrinsic defense mechanism, which is operational after birth. It is equipped with the capacity to identify foreign substances or pathogens by the proteins present on the surface of immune cells, thereby distinguishing them from self-tissues. In instances where unidentified substances elude immune surveillance, an inflammatory response is triggered to eliminate these potential threats. During the inflammatory response, monocytes differentiate into macrophages, amplifying their phagocytic activity and the level of reactive oxygen species within cells, thereby facilitating the eradication of pathogens. Consequently, moderate inflammation plays a pivotal role in enhancing immune system function. However, excessive stimulation and activation of the immune system can have detrimental consequences on bodily functions. During such periods, monocytes undergo mass differentiation into macrophages, which in turn produce copious amounts of cytokines that stimulate excessive inflammatory responses. This can result in cytokine storms, leading to severe tissue inflammation, organ damage or failure, and, in certain cases, cell carcinogenesis.

CD69, CD86, CD80, and CD82 are transmembrane proteins that are present on the surface of activated immune cells. CD69 expression is generally regarded as an early activation marker of immune cells, while CD80 is found on dendritic cells, activated B cells, and monocytes, and CD86 is present on antigen-presenting cells; CD80 and CD86 stimulate T-cell activation, differentiation, and proliferation. CD82, a transmembrane protein, has been found to be expressed at higher levels in human monocytes and macrophages [67], and has been shown to induce inflammation by promoting monocyte differentiation into macrophages [68]. NF-κB and AP-1 have been identified as key regulators of DNA transcription, cytokine production, and cell survival [67].

**Figure 9:**
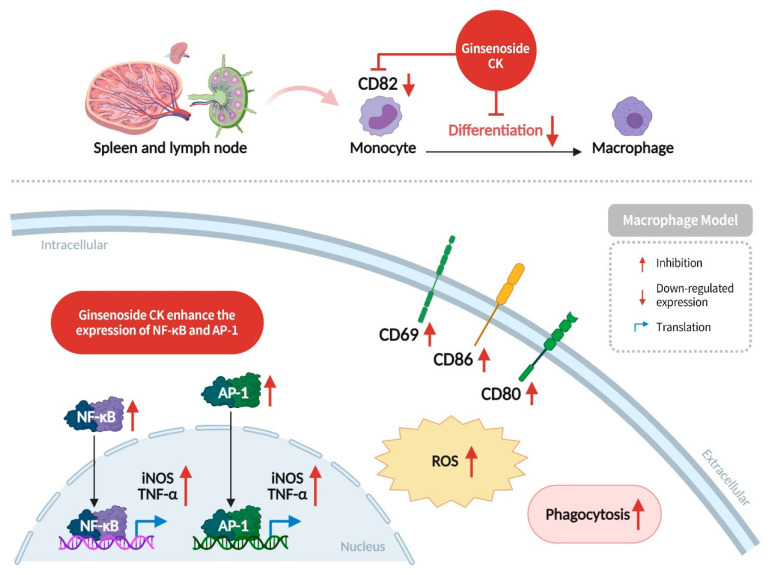
Ginsenoside CK can enhance the expression of CD69, CD80, and CD86, stimulate the NF-κB and AP-1 inflammatory signaling pathway, and induce macrophages to secrete iNOS and TNF-α to enhance inflammatory response. Conversely, CK can also inhibit the expression of CD82 protein, reducing monocyte differentiation into macrophages to avoid excessive inflammatory responses.

Research has demonstrated that ginsenoside CK enhances the expression of CD69, CD80, and CD86, thereby activating the immune surveillance capabilities of macrophages. In addition, it has been shown to stimulate NF-κB and AP-1 inflammatory signaling pathways, as well as inducing macrophages to secrete iNOS and TNF-α, thus enhancing the inflammatory response [69]. Conversely, ginsenoside CK has also been observed to inhibit the expression of the CD82 protein, thereby reducing monocyte differentiation into macrophages (Figure 9) [69]. This bidirectional regulation of macrophage phagocytosis and differentiation by ginsenoside CK has been demonstrated to prevent excessive inflammatory responses, thereby reducing the likelihood of normal cells turning cancerous, achieving immunomodulatory effects.

### 2.7. Activation of Apoptotic Proteases by Ginsenoside CK Inducing Cancer Cell Apoptosis

Apoptosis, a programmed process of cell death, is vital for the maintenance of cellular metabolism by regulating the balance between cell proliferation and death. This process eliminates damaged, senescent, or unnecessary cells, thus ensuring the stability of internal cellular environments and the normal functioning of physiological systems. The dysregulation of apoptosis can result in various diseases, including autoimmune diseases, neurodegenerative disorders, and cancer [70]. Cancer cells can inhibit apoptosis, allowing them to continuously proliferate and affect the survival of other normal cells. Therefore, inducing apoptosis in cancer cells can effectively kill them.

**Figure 10:**
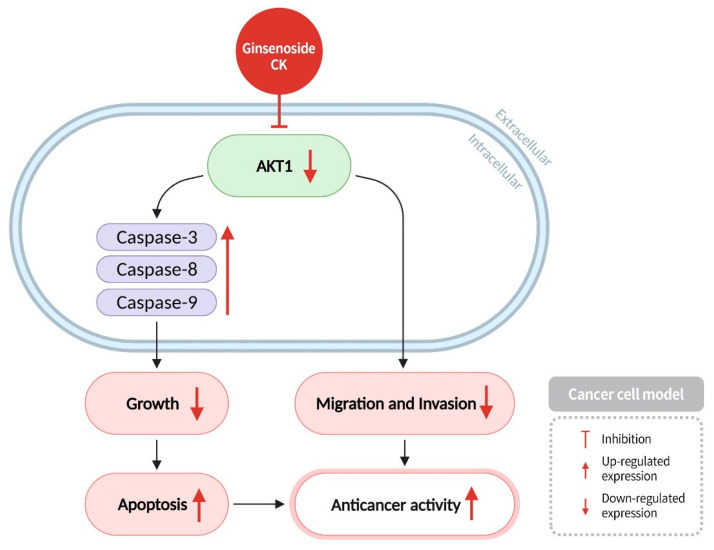
Ginsenoside CK can inhibit the activity of AKT1 in cancer cells, increase the expression of apoptotic proteases Caspase-3, Caspase-8, and Caspase-9, thereby inducing cancer cell apoptosis. Additionally, it also suppresses the migration and invasion of cancer cells.

AKT1, also known as protein kinase B-1, plays a crucial role in cell proliferation, apoptosis, migration, and glucose metabolism. It promotes cell survival and inhibits the activity of apoptotic proteases [63]. Caspases are cysteine proteases and specific endoproteases that can hydrolyze cysteine residues in proteins and cleave peptide bonds linked to aspartic acid residues to activate other proteases. Hence, the activation of caspases usually results in a cascade of protein hydrolysis. Although the caspase-mediated process can lead to the inactivation of cell-related substrates, it can also participate in the signaling processes of apoptosis and inflammation [71]. Caspases play an important role in apoptosis and inflammation, with Caspase-3 being the terminal executor of apoptosis [72]. When cells receive apoptosis signals, Caspase-8 and Caspase-9 stimulate the activation of Caspase-3, initiating a series of reactions that lead the cell into apoptosis and resulting in cell death. In addition, studies have shown that ginsenoside CK can inhibit the activity of AKT1 in cancer cells, thereby increasing the expression levels of the apoptotic proteases Caspase-3, Caspase-8, and Caspase-9, and inducing apoptosis in cancer cells. Concurrently, ginsenoside CK suppresses cancer cell migration and invasion, thus achieving anticancer effects (Figure 10) [73,74].

### 2.8. Enhancement of Mitochondrial Reactive Oxygen Species (ROS) by Ginsenoside CK Inducing Cancer Cell Apoptosis

Reactive oxygen species (ROS) have been identified as playing a crucial role in the pathogenesis of chronic human diseases and in various treatment modalities, such as chemotherapy and radiotherapy [75]. ROS can originate from both external and internal sources. External sources include ionizing radiation, UV radiation, smoking, air pollution, and chemotherapy drugs, while internal sources generate ROS through the electron transport chain. Excessive ROS can cause a loss in mitochondrial membrane potential, preventing cells from producing sufficient energy, and can lead to oxidative stress-induced cell damage and even cell death [76].

**Figure 11:**
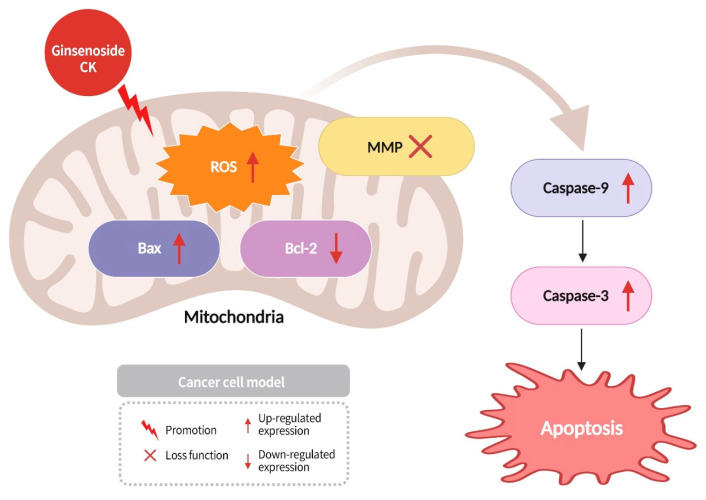
Ginsenoside CK enhances the concentration of mitochondrial ROS in cancer cells, leading to a loss in mitochondria membrane potential and increased expression of Bax, Caspase-3, and Caspase-9, while decreasing the expression of Bcl-2, thus inducing apoptosis in cancer cells.

Cancer cells can regulate the concentration of ROS within themselves by increasing the expression of antioxidant enzymes, controlling ROS levels to avoid cell death. This delicate balance is crucial for the survival of cancer cells; disrupting this balance can effectively lead to cancer cell death. The balance between pro- and anti-apoptotic proteins is critical for cell survival. Bcl-2, an anti-apoptotic protein, and Bax, a pro-apoptotic protein, are two such proteins that regulate apoptosis in cells. Bcl-2 inhibits apoptosis, while the expression of Bax promotes apoptosis [77]. Caspases represent a family of cysteine proteases, which are distinct from other proteases in that they can hydrolyze cysteine residues in proteins and cleave peptide bonds linked to aspartic acid residues, thus activating other proteases [77]. Specifically, Caspase-9 and Caspase-3 are the main enzymes in the apoptosis signaling pathway and have been demonstrated to induce the occurrence of apoptosis. Research has found that ginsenoside CK can increase the concentration of ROS in the mitochondria of cancer cells, leading to the loss of mitochondrial membrane potential (MMP). It also enhances the expression of pro-apoptotic protein Bax and apoptotic proteases Caspase-3 and Caspase-9, while reducing the expression of anti-apoptotic protein Bcl-2, ultimately promoting apoptosis in cancer cells (Figure 11) [74].

### 2.9. Ginsenoside CK Enhances Sensitivity to Tumor Necrosis Factor-Related Apoptosis-Inducing Ligand (TRAIL) to Induce Cancer Cell Apoptosis

Tumor necrosis factor-related apoptosis-inducing ligand (TRAIL) is a member of the tumor necrosis factor superfamily (TNFSF) that has the capacity to induce cellular inflammation, apoptosis, proliferation, and differentiation. In addition, it has the ability to regulate immune cells, inhibit viral replication, and prevent the formation of cancer cells. Literature reviews confirm that various cancer cells are particularly sensitive to apoptotic signals induced by TRAIL, whereas most normal cells exhibit resistance to TRAIL [78]. This is because TRAIL possesses specific toxicity against cancer cells, capable of inducing apoptosis in tumor cells [79]. Hence, TRAIL possesses the potential to be developed into a promising anticancer drug.

DR5 is a receptor for TRAIL that is present on the surface of cells. TRAIL activates through interaction with DR5, driving the formation of death-inducing signaling complexes that trigger apoptosis signaling responses. The loss of mitochondrial membrane potential, induced by ROS, can impede the generation of energy by cells, consequently leading to oxidative stress and cell damage, ultimately resulting in autophagy and cell death. Autophagy, a pivotal cellular process involved in the elimination of toxic proteins and foreign pathogens, plays a critical role in the pathogenesis of infections, aging, and human diseases [80]. The p53 protein has the capacity to regulate apoptosis, cellular senescence, and inhibit angiogenesis [81].

**Figure 12:**
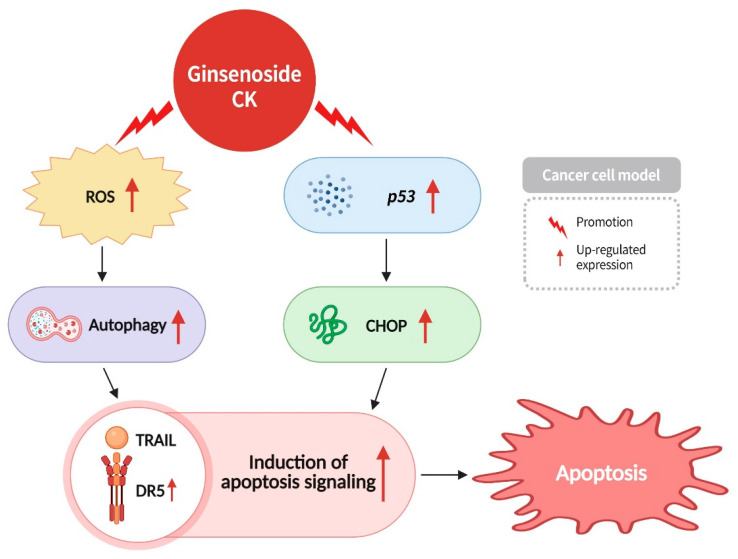
Ginsenoside CK can induce autophagy in cancer cells by increasing ROS concentration. It can also enhance the expression of tumor suppressor gene *p53* and CHOP protein, promoting the expression of DR5, thereby increasing the sensitivity of cancer cells to TRAIL. This further induces apoptosis signaling, ultimately resulting in cancer cell death.

CHOP, a nucleoprotein and a transcription factor, has been shown to trigger endoplasmic reticulum stress-induced apoptosis, and to bind to the DR5 promoter, enhancing DR5 expression [82]. According to studies, ginsenoside CK can induce autophagy in cancer cells by increasing ROS levels and can also increase the expression of the tumor suppressor gene p53 and CHOP proteins. This, in turn, has been shown to enhance cancer cell sensitivity to TRAIL, leading to apoptosis signaling and inducing cancer cell apoptosis (Figure 12) [82]. In the development of TRAIL-based anticancer drugs, ginsenoside CK is undoubtedly an important candidate.

### 2.10. Enhanced T Cell Recognition by Ginsenoside CK for Cancer Cell Elimination

Immune checkpoints consist of the PD-1 receptor on the surface of T cells and PD-L1 on the surface of autologous cells. When the PD-1 on T cells fails to recognize PD-L1 on normal cells, it categorizes them as foreign entities that must be eliminated [83]. However, to avoid immune system attacks, cancer cells not only produce but also increase the expression of PD-L1 on their surface. When T cells with PD-1 receptors inspect the area, PD-1 on the T-cell binds to PD-L1 on the cancer cell, suppressing the T-cell’s immune response, allowing the cancer cell to pass immune surveillance. Thus, being able to block the binding of PD-L1 on cancer cells to the PD-1 receptor on T cells would then stimulate T cells to initiate an immune response, inducing cancer cell death.

**Figure 13:**
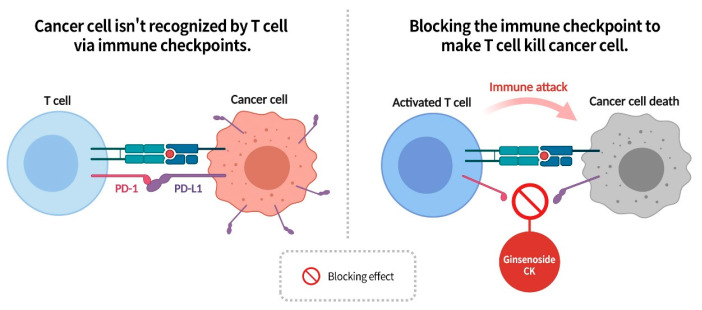
Ginsenoside CK can block the binding of T cell PD-1 with cancer cell PD-L1, preventing cancer cells from evading immune system surveillance and enhancing T cell recognition of cancer cells.

In recent years, PD-1/PD-L1 checkpoint inhibitors have garnered significant attention in immunotherapy for cancer due to their lower toxicity and more durable treatment effects compared to other tumor immunotherapies [84]. Research has shown that ginsenoside CK can block the interaction between PD-1 on T cells and PD-L1 on cancer cells, preventing cancer cells from evading immune surveillance by producing PD-L1 cells. This enhances T cell recognition capability, enabling the immune system to successfully attack and eliminate cancer cells (Figure 13) [85]. In the context of PD-1/PD-L1 immune checkpoint inhibitor therapy for cancer, the effectiveness of ginsenoside CK deserves spotlight attention, akin to the results observed from CAR-T therapy.

**Table 3:** The summarize table of in vitro anticancer mechanisms of ginsenoside CK.

Types of Treatment	Section No./ Test Model	Function	Mode of Action	Cell Line/Test Dosage	IC_50_ for only CK
The effects on cancer cells themselves	Section 2.1/Human osteosarcoma cells	Inhibit malignant tumor proliferation and invasion	CK inhibits the PI3K/mTOR/p70S6K1 signaling pathway in cancer cells and reduces the expression of MMP.	MG-63, U2-OS/ 20 μM	20 μM
	Section 2.2/ Rat glioma cells	Inhibit cancer cell migration	CK can downregulate the SDF-1/CXCR4 signaling pathway in cancer cells, thereby inhibiting the expression of PKCα and ERK protein phosphorylation, reducing the expression of MMP.	C6/ 1 μM	3–10 μM
	Section 2.4/Human monocytic leukemia cells	Inhibits telomerase activity in cancer cells	CK inhibits the activity of telomerase reverse transcriptase in cancer cells, ultimately leading to cancer cell death.	U937/ 48 μM	none
	Section 2.7/Human breast cancer cells	Enhance the expression of apoptosis proteins and induce apoptosis of cancer cells	CK inhibits AKT1 activity, activates the expression of apoptotic proteases Caspase-3, Caspase-8, and Caspase-9, induces cancer cell apoptosis, and also inhibits cancer cell migration and invasion.	SKBR3/ 50 μM	50 μM
	Section 2.8/Human neuroblastoma	Increase the ROS content in cancer cell mitochondria and induce apoptosis of cancer cells	CK increases the ROS concentration in cancer cell mitochondria, causing the cancer cell mitochondria to lose membrane potential, increasing the expression of pro-apoptotic protein Bax and apoptotic protease Caspase-3/9 and reducing the expression of anti-apoptotic protein Bcl-2.	SK-N-SH/ 15 μM	15 μM
	Section 2.9/Human colon cancer cells	Enhance the sensitivity of cancer cells to TRAIL-induced apoptosis	CK increases the concentration of ROS, causing autophagy in cancer cells and increasing the expression of the p53 gene, which can promote the expression of DR5, thereby increasing the sensitivity of cancer cells to TRAIL. DR5 and TRAIL combine to promote cell apoptosis.	HCT116/ 50 μM	>50μM
Angiogenesis inhibition	Section 2.3/Human umbilical vein endothelial cells	Anti-angiogenesis	CK inhibits the p38 and AKT cell pathways downstream of PI3K, blocking angiogenesis.	HUVECs/ 50 μM	85 μM
Modulation of immune response to identify cancer cell	Section 2.6/Mouse macrophages	Regulate immune activity	CK upregulates the expression of CD69, CD80 and CD86 proteins to activate macrophage function, enhance macrophage immune detection ability, stimulate the expression of NF-κB and AP-1 inflammatory signaling pathways.	RAW264./ 48 μM	49–64 μM
	Section 2.10/PD-1 and PD-L1 proteins	Enhance the ability of T cells to recognize cancer cells	CK can block the binding of T cell PD-1 to cancer cell PD-L1, thus improving the ability of T cells to recognize cancer cells.	ELISA of PD-1and PD-L1/ 0.1 μM	none
Inflammation regulation	Section 2.5/Mouse macrophages	Suppress excessive inflammation	CK inhibits LPS-induced inflammatory responses, reduces the expression of ERK and JNK mitogen-activated protein kinases, and reduces the production of NO and PGE2 that can cause cell inflammation.	RAW264.7/40 μM	41–60 μM
	Section 2.6/Mouse macrophages	Regulate immune activity	CK can inhibit the expression of CD82 protein and reduce the differentiation of monocytes into macrophages.	U937/ 48 μM	none

## 3. Conclusions

Current cancer therapies are principally concerned with the suppression of cancer cell proliferation and metastasis, the inhibition of angiogenesis, and the modulation of immune responses. When the physical removal of tumors is feasible, this is also pursued. It is imperative to prevent chronic inflammation to avoid cellular transformation into malignancies. In this review, we summarize that ginsenoside compound K (CK) suppresses cancer cell proliferation by modulating the PI3K signaling pathway and metastasis through downregulation of MMP activity. It has also been demonstrated that the drug promotes apoptosis by reducing AKT1 activity, enhancing cancer cell sensitivity to TRAIL, increasing ROS levels, and disrupting mitochondrial membrane potential (MMP). Furthermore, CK has been shown to inhibit telomerase activity, a process that contributes to the senescence and death of cancer cells due to telomere shortening. CK has also been shown to inhibit angiogenesis by regulating p38 and AKT of the PI3K signaling pathway. CK has been shown to impede the interaction between PD-1 on T cells and PD-L1 on cancer cells, thereby restoring immune checkpoint surveillance. Furthermore, it has been demonstrated that the expression of CD69, CD80, and CD86 on macrophages is enhanced, thereby improving immune recognition. CK has been demonstrated to reduce CD82 expression on macrophages, thereby constraining the differentiation of monocytes into macrophages. Additionally, CK has been shown to curtail the inflammatory production of NO and PGE2 by inhibiting the expression of ERK and JNK. CK has been demonstrated to inhibit NLRP3 inflammasome activation in renal cells via mitochondrial-related signaling pathways. The regulation of CK for NLRP3 constitutes a pivotal mechanism underlying its anti-inflammatory effects. CK has been demonstrated to regulate immunity in a bidirectional manner. In recent years, there has been a growing trend of promoting whole foods and natural ingredients. As a result, natural ingredients derived from plants may be more readily accepted than chemically synthesized substances. Consequently, CK, a byproduct of the biotransformation of ginsenoside components, has garnered increasing attention, accompanied by a proliferation of related research publications in prominent international journals. However, there is still a paucity of research on the cellular mechanisms of CK in treating cancer. This article delves into the mechanism of ginsenoside CK on cancer cells in vitro and employs graphical representations to elucidate the intricate interactions among cells. In the future, it is our objective to further explore the research results of CK on cancer in vivo. We hypothesize that we will find a correlation between in vivo treatment and in vitro experiments in cellular mechanisms. We also hypothesize that we will clarify the drug mechanism and promote drug development.

## Figures and Tables

**Table 1:** Summary of safe doses and NOAELs in current preclinical toxicity studies of ginsenoside CK.

Type	Model	Administration	Dosing Frequency	Administrated Period (Weeks)	Dose (mg/kg)	MED * (mg/kg)
acute	mice	oral	single	2	10,000 (NOAEL)	10,000
acute	rat	oral	single	2	8000 (NOAEL)	12,800
chronic	rat (male)	oral	once a day	26	40 (NOAEL)	64
chronic	dog	oral	once a day	26	12 (NOAEL)	57.6
subchronic	dog	i.v. injection	once a day	13	6.7 (NOAEL)	32.2

* MED (Mouse equivalent dose) = dose of different model × dose conversion factor; dose conversion factor of rat into mouse is 1.6 and dog into mouse is 4.8.

**Table 2:** Summary of the effective therapeutic doses of ginsenoside CK against different types of cancer in mouse models.

Carcinoma	Cell Line	Administration	Dosing Frequency	Administrated Period	Dose (mg/kg)	Tumor Shrink (%)
Hepatoma	BEL7402	i.p. injection	once two days	5 weeks	10	86 (weight)
Hepatoma	Hep-G2	i.p. injection	once three days	18 days	10	50 (volumn)
Lung	A549 cells	i.v. injection	once three days	15 days	15	41 (volumn)
Breast	MCF10DCIS	i.p. injection	once two days	3 weeks	1	63 (volumn)
Neuroblastoma	SK-N-BE(2)	i.p. injection	three times a week	53 days	30	56 (weight)
Leukemia	C1498, FLT3+	i.p. injection	once two days	10 days	5	56, 54 (WBC count)
Colorectal	AOM/DSS-induced	oral	freely fed	6 weeks	60	79 (volumn)
Tumor shrink(%) = [1 − (Tumor size(CK)/Tumor size(con.))] × 100%						

## Abbreviations

The following abbreviations are used in this manuscript:

CK	Ginsenoside CK
IARC	International Agency for Research on Cancer
PPD	Propanediol
PPT	Propanetriol
MMP	Matrix metalloproteinase
SDF-1	Stromal cell-derived factor 1
MAPK	Mitogen-activated protein kinase
ERK	Extracellular signal regulated kinase
bFGF	Basic fibroblast growth factor
VEGF	Vascular endothelial growth factor
hTERT	Human telomerase reverse transcriptas
